# Frequency and clinical impact of *CDKN2A/ARF/CDKN2B* gene deletions as assessed by in-depth genetic analyses in adult T cell acute lymphoblastic leukemia

**DOI:** 10.1186/s13045-018-0639-8

**Published:** 2018-07-24

**Authors:** E. Genescà, A. Lazarenkov, M. Morgades, G. Berbis, N. Ruíz-Xivillé, P. Gómez-Marzo, J. Ribera, J. Juncà, A. González-Pérez, S. Mercadal, R. Guardia, M. T. Artola, M. J. Moreno, J. Martínez-López, L. Zamora, P. Barba, C. Gil, M. Tormo, A. Cladera, A. Novo, M. Pratcorona, J. Nomdedeu, J. González-Campos, M. Almeida, J. Cervera, P. Montesinos, M. Batlle, S. Vives, J. Esteve, E. Feliu, F. Solé, A. Orfao, J. M. Ribera

**Affiliations:** 1 Josep Carreras Leukaemia Research Institute (IJC), Campus ICO-Germans Trias i Pujol, Universitat Autònoma de Barcelona (UAB), Badalona, Spain; 2 Clinical Hematology Department, ICO-Hospital Germans Trias i Pujol, Badalona, Universitat Autònoma de Barcelona (UAB), Barcelona, Spain; 3 Institute for Research in Biomedicine (IRB Barcelona), The Barcelona Institute of Science and Technology, Research Program on Biomedical Informatics, Universitat Pompeu Fabra, Barcelona, Spain; 4 Clinical Hematology Service, Hospital Duran i Reynals-ICO, Hospitalet del LLobregat, Barcelona, Spain; 5 Clinical Hematology Service, Hospital Josep Trueta-ICO, Girona, Spain; 6 Clinical Hematology Service, Hospital Universitario de Donostia, Donostia, Spain; 7 Clinical Hematology Service, Hospital Vírgen de la Victoria, Málaga, Spain; 8 Hematology Department, Hospital 12 de Octubre, CNIO, Universidad Complutense, Madrid, Spain; 9 Clinical Hematology Service, Hospital Universitari de la Vall d’Hebron, Barcelona, Spain; 10 Clinical Hematology Service, Hospital General de Alicante, Alicante, Spain; 11 Clinical Hematology Service, Hospital Clínico de Valencia, Valencia, Spain; 12 Clinical Hematology Service, Hospital Son Llàtzer, Palma, Spain; 13 Clinical Hematology Service, Hospital Son Espases, Palma, Spain; 14 Clinical Hematology Service, Hospital de la Santa Creu i Sant Pau, Barcelona, Spain; 15 Clinical Hematology Service, Hospital Vírgen del Rocío, Sevilla, Spain; 16 Banco Nacional de ADN Carlos III, Universidad de Salamanca, Salamanca, Spain; 17 Biobanco de la Fe, Instituto de Investigación Sanitaria La Fe (IIS La Fe), Valencia, Spain; 18 Clinical Hematology Service, Hospital La Fe, Valencia, Spain; 19 Clinical Hematology Service, Hospital Clínic de Barcelona, Barcelona, Spain; 20 Centro de Investigación del Cáncer (IBMCC-CSIC/USAL) (CIC), Hospital Clínico Universitario de Salamanca (HUS), Instituto Bio-Sanitario de Salamanca (IBSAL), CIBERONC, Salamanca, Spain; 21 ALL Research Group, Josep Carreras Leukaemia Research Institute (IJC), Camí de les Escoles s/n. Edifici IJC, 08916 Badalona, Spain

**Keywords:** T-ALL, *CDKN2A/ARF*, *CDKN2B*, Prognosis, MRD

## Abstract

Recurrent deletions of the *CDKN2A/ARF/CDKN2B* genes encoded at chromosome 9p21 have been described in both pediatric and adult acute lymphoblastic leukemia (ALL), but their prognostic value remains controversial, with limited data on adult T-ALL. Here, we investigated the presence of homozygous and heterozygous deletions of the *CDKN2A/ARF* and *CDKN2B* genes in 64 adult T-ALL patients enrolled in two consecutive trials from the Spanish PETHEMA group. Alterations in *CDKN2A/ARF/CDKN2B* were detected in 35/64 patients (55%). Most of them consisted of 9p21 losses involving homozygous deletions of the *CDKNA/ARF* gene (26/64), as confirmed by single nucleotide polymorphism (SNP) arrays and interphase fluorescence in situ hybridization (iFISH). Deletions involving the *CDKN2A/ARF/CDKN2B* locus correlated with a higher frequency of cortical T cell phenotype and a better clearance of minimal residual disease (MRD) after induction therapy. Moreover, the combination of an altered copy-number-value (CNV) involving the *CDKN2A/ARF/CDKN2B* gene locus and undetectable MRD (≤ 0.01%) values allowed the identification of a subset of T-ALL with better overall survival in the absence of hematopoietic stem cell transplantation.

**Electronic supplementary material:**

The online version of this article (10.1186/s13045-018-0639-8) contains supplementary material, which is available to authorized users.

At present, treatment response based on minimal residual disease (MRD), monitoring for early and accurate identification of high-risk patients in whom treatment might be intensified, represents a milestone in virtually all childhood and adult acute lymphoblastic leukemia (ALL) trials [1, 2]. Despite this, more extended molecular analyses performed at diagnosis in ALL have also proven to contribute to the identification of ALL subtypes that respond better to specific targeted therapies and to refine the classical risk-stratification schemes used at baseline [3]. However, from all genomic markers identified so far [4], only a few are routinely used for the clinical management of ALL, particularly in T cell ALL (T-ALL). This is due to the still limited data available about their frequency and independent prognostic impact, in large cohorts of T-ALL patients homogeneously treated in the MRD era.

Here, we investigated the presence and frequency of copy-number-value alterations (CNA) at chromosome 9p21 which involved the *CDKN2A/ARF* and *CDKN2B* genes in a cohort of 64 adult T-ALL patients enrolled in two consecutive Spanish PETHEMA (Programa Español para el Tratamiento de Hemopatías Malignas) trials (details about the patient cohort are available in Additional file 1: Figure S2 and Table S4), using a genomic quantitative polymerase chain reaction (qPCR) technique [5, 6] (Additional file 1: Table S1). An overall frequency of CNA at chromosome 9p21 of 55% (35/64 cases) was observed, 20% of the cases (13/64) showing a discrepant CNA profile for the *CDKN2A/ARF* and *CDKN2B* genes. Of note, the CNA values identified by qPCR were fully concordant with those obtained by SNP-arrays and iFISH analyses, once qPCR CNA values had been corrected for the contamination by normal DNA-diploid cells in the sample (Additional file 1: Table S2).

A significant association was observed between the presence of (bi- or mono-allelic) deletion of the *CDKN2A/ARF/CDKN2B* genes and cortical T-ALL (T-III, according to the EGIL criteria) [7] (47%), while this molecular alteration was found at very low frequency in the early T cell precursor ALL group [8] (ETP-ALL, T-I or Pro-T according to the EGIL criteria) (3%; *p* = 0.002). Adult T-ALL patients showing (bi- or mono-allelic) deletions of *CDKN2A/ARF* had deeper MRD responses (MRD levels ≤ 0.1%) than those who had normal copy-number-values (CNV) (90 vs. 68% of cases, *p* = 0.04) (Table 1), while there was a trend for *CDKN2B* gene deletions (89 vs 71%; *p* = 0.11). When deletion of the *CDKN2A/ARF* and/or *CDKN2B* genes were considered together, differences were statistically significant (91% *vs* 65%, *p* = 0.02). This is due to the fact that among the *CDKN2B* non-deleted cases, some patients with deletions of the *CDKN2A* gene were included. Therefore, the identification of a pure *CDKN2A/ARF* and *CDKN2B* non-deleted group of patients allowed for a better discrimination between good and poor responders. This association was even more clear when we considered an MRD threshold of ≤ 0.01% (Table 1). In addition, no patient with (bi- or mono-allelic) *CDKN2A/ARF/CDKN2B* deletions required second induction therapy due to poor morphologic and/or MRD response, while 32% of patients harboring normal diploid CNV (two copies of the *CDKN2A/ARF/CDKN2B* genes) did (*p* = 0.001). Of note, most patients (6/8, 75%) who showed two copies of the *CDKN2A/ARF/CDKN2B* genes in their blast cells, and required intensification of induction therapy, had ETP-ALL (*p* < 0.001), such cases corresponding to 6/10(60%) ETP-ALL cases in the cohort.

**Table 1:** Association between the CNA status for the *CDKN2A/ARF/CDKN2B* locus and early response to treatment as assessed by the MRD levels detected at the end of induction therapy

*CDN2A/ARF/CDKN2B* gene status	MRD ≤ 0.1%	*P* value	MRD ≤ 0.01%	*P* value
*CDKN2A/ARF*				
Bi or mono-allelic deletion (*n* = 30)	27 (90)	0.04	22 (73)	0.03
No deletion (*n* = 30)	17 (68)		11 (44)	
*CDKN2B*				
Bi or mono-allelic deletion (*n* = 27)	24 (89)	0.10	19 (70)	0.12
No deletion (*n* = 28)	20 (71)		14 (50)	
*CDKN2A/ARF* and/or *CDKN2B*				
Bi or mono-allelic deletion (*n* = 32)	29 (91)	0.02	24 (75)	0.007
No deletion (*n* = 23)	15 (65)		9 (39)	

Results expressed as number of cases (percentage)

As a consequence of their better response to induction treatment, most patients with an altered *CDKN2A/ARF/CDKN2B* CNV (32/34, 94%) did not require an allogeneic-hematopoietic stem cell transplantation (allo-HSCT) according to the treatment protocol, in contrast to 11/28 patients (40%) with a normal diploid *CDKN2A/ARF/CDKN2B* genotype (*p* = 0.001).

Overall survival (OS) analysis based on the *CDKN2A/ARF/CDKN2B* copy-number status, MRD data [9] and treatment with a transplant, as prognostic factors, allowed the identification of a subgroup of patients with a very good prognosis which showed mono or bi-allelic deletions of the *CDKN2A/ARF/CDKN2B* genes and MRD levels ≤ 0.01% (3y OS probability of 75% [56–94%]) vs. only 36% [8–64%]), for the remaining patients; (*p* = 0.05), when the OS was censored at transplantation (Fig. 1a); of note, the significance of the differences was lower (*p* = 0.1) when patients’ follow-up was not censored at transplantation (Fig. 1b).

**Fig. 1:**
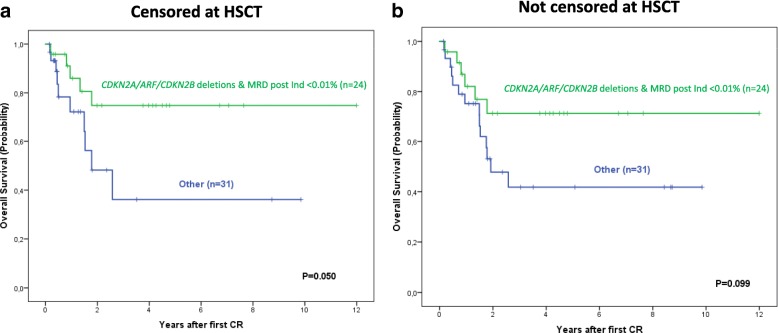
Prognostic impact of the *CDKN2A/ARF/CDKN2B* gene CNV status and MRD levels (≤ 0.01 %) on overall survival (OS) of adult T-ALL patients (*n* = 62). **a** OS for patients with follow-up censored at allo-HSCT. **b** The OS without censoring at allo-HSCT

When we searched for independent prognosis factors for OS, we observed that despite deletions of the *CDKN2B* gene (particularly mono-allelic *CDKN2B* gene deletions), but not the *CDKN2A/ARF* gene deletions, conferred a better prognosis in terms of OS (3y OS probability of 63% [43–83%] vs.37% [18–55%], *p* = 0.045) (Additional file 1: Figure S1A-B) in the univariate analysis, MRD after induction therapy was the only variable with an independent predictive value for OS in the multivariate analysis (Additional file 1: Table S3). Our results are in line with the findings reported by Liu et al. [10], but need to be validated in a larger and independent cohort of adult T-ALL. Recently, it has been highlighted the importance of *NOTCH I/FBXW7* and *N/K RAS*/ *PTEN* point mutations in the OS of adult T-ALL patients [3], therefore would be interesting to assess the impact of these point mutations in our cohort in combination, or not, with *CDKN2B* deletions.

In summary, here, we confirm the high frequency of (mono and bi-allelic) deletions of the *CDKN2A/ARF/CDKN2B* genes also in adult T-ALL, and highlight the specific association between the loss of the *CDKN2A/ARF* and *CDKN2B* genes and a better response to therapy and prolonged OS, respectively. More importantly, identification of CNA in the *CDKN2A/ARF/CDKN2B* gene locus, together with the MRD levels at the end of induction, contributed to the identification of a subgroup of T-ALL patients in whom intensification of therapy with an allo-HSCT might not be of great clinical benefit.

## Additional file

Additional file 1: **Table S1.** Frequency and type of CDKN2A/ARF/CDKN2B gene deletions as detected by qPCR in adult T-ALL (*n* = 64). **Table S2.** Comparison between the CNA status of the *CDKN2A/ARF* and *CDKN2B* genes in adult T-ALL as assessed by qPCR, SNP-array and iFISH techniques. **Table S3.** Adult T-ALL: prognostic factors for overall survival. **Table S4.** Adult T-ALL patient characteristics at diagnosis and follow-up. **Table S5.** (A) RCN values obtained for the *CDKN2A/ARF* and *CDKN2B* genes in selected samples with a 100% blast cell content. (B). Most robust cut-off values to distinguish between normal, heterozygous and homozygous genotypes. The mean and standard deviation (SD) of the values obtained in panel A are indicated for each genotype. **Figure S1.** Prognostic impact of the CDKN2B gene CNA status on overall survival of adult T-ALL patients (*n* = 62). In panel A all CDKN2B gene deletions were analyzed together, while in panel B bi-allelic and mono-allelic CDKN2B gene deletions were separately considered. **Figure S2.** Flowchart summarizing the HR-20011 PETHEMA treatment protocol, including the time points at which MRD assessment was performed (highlighted in red). **Figure S3.** Calibration curves used to calculate RCN values according to the different percentage contamination of the sample by normal (i.e. non-blastic) cells. In panel A, a pure (100% blasts) homozygous sample was mixed with different amounts of normal (2 N) DNA, as shown on the x-axis. RCN values are shown on the y-axis. In panel B a pure (100%) heterozygous sample was mixed with different amounts of normal (2 N) DNA, as shown on the x-axis; RCN values are depicted on the y-axis. (PDF 274 kb)

## Abbreviations

ALL: Acute lymphoblastic leukemia
Allo-HSCT: Allogeneic-hematopoietic stem cell transplantation
BM: Bone marrow
CNA: Copy number alterations
CNV: Copy-number-value
CR: Complete remission
EGIL: European Group for the Immunological Classification of Leukemias
ETP-ALL: Early T cell precursor acute lymphoblastic leukemia
iFISH: Interphase fluorescence in situ hybridization
MRD: Minimal residual disease
OS: Overall survival
PETHEMA: Programa Español para el Tratamiento de Hemopatías Malignas
qPCR: Quantitative polymerase chain reaction
RCN: Relative copy number
SD: Standard deviation
SNP-array: Single nucleotide polymorphism array
T-ALL: T cell acute lymphoblastic leukemia
WBC: White blood cell count
WHO: World Health Organization
