# Supplementary material for: Frequency and clinical impact of CDKN2A/ARF/CDKN2B gene deletions as assessed by in-depth genetic analyses in adult T cell acute lymphoblastic leukemia
Source: J Hematol Oncol. 2018 Jul 24;11:96. doi: 10.1186/s13045-018-0639-8 (PMC6057006; doi:10.1186/s13045-018-0639-8)
Supplement: Supplementary file 1 — Table S1. Frequency and type of CDKN2A/ARF/CDKN2B gene deletions as detected by qPCR in adult T-ALL (n = 64). Table S2. Comparison between the CNA status of the CDKN2A/ARF and CDKN2B genes in adult T-ALL as assessed by qPCR, SNP-array and iFISH techniques. Table S3. Adult T-ALL: prognostic factors for overall survival. Table S4. Adult T-ALL patient characteristics at diagnosis and follow-up. Table S5. (A) RCN values obtained for the CDKN2A/ARF and CDKN2B genes in selected samples with a 100% blast cell content. (B). Most robust cut-off values to distinguish between normal, heterozygous and homozygous genotypes. The mean and standard deviation (SD) of the values obtained in panel A are indicated for each genotype. Figure S1. Prognostic impact of the CDKN2B gene CNA status on overall survival of adult T-ALL patients (n = 62). In panel A all CDKN2B gene deletions were analyzed together, while in panel B bi-allelic and mono-allelic CDKN2B gene deletions were separately considered. Figure S2. Flowchart summarizing the HR-20011 PETHEMA treatment protocol, including the time points at which MRD assessment was performed (highlighted in red). Figure S3. Calibration curves used to calculate RCN values according to the different percentage contamination of the sample by normal (i.e. non-blastic) cells. In panel A, a pure (100% blasts) homozygous sample was mixed with different amounts of normal (2 N) DNA, as shown on the x-axis. RCN values are shown on the y-axis. In panel B a pure (100%) heterozygous sample was mixed with different amounts of normal (2 N) DNA, as shown on the x-axis; RCN values are depicted on the y-axis. (PDF 274 kb) [file 13045_2018_639_MOESM1_ESM.pdf]

On line Supplemental Material and Methods

Patients and samples

Sixty-four newly diagnosed T-ALL cases, from which 62 had available clinical data were included in this study. Forty-three were males and 19 females, with a median age (range) of 36 years (16-72). T-ALL diagnosis was established locally according to World Health Organization (WHO) 2008 criteria, and the criteria proposed by Zurbier *et al* were used to define ETP-ALL (12/61, 20%) [6]. The EGIL (European Group for the Immunological Classification) criteria [7] were used to define the immunologic subtype of T-ALL by central review of immunophenotypic data and reports. Patients were treated according to two consecutive MRD-oriented high-risk adult ALL PETHEMA protocols: ALL-HR-2003 (NCT00853008) and ALL-HR-11 (NCT01540812) (still ongoing). Most T-ALL patients were included in these two protocols, with the exception of those with cortical T-ALL with white blood cell (WBC) counts $<100x10^9$/L who were considered as standard-risk ALL, and those aged >60 years (n=4), who were included in a protocol for elderly patients (ALL07OLD). Response to induction chemotherapy was evaluated by morphologic and flow cytometric studies of bone marrow. Good responders (<5% blasts and MRD levels ≤0.1%) proceeded to consolidation and maintenance chemotherapy. Poor responders (>5%blasts and/or MRD ≥0.1%) received intensification of induction treatment, followed by Allo-HSCT. As shown in Supplemental Figure S2, MRD assessment by flow cytometry was partially centralized in the ALL-HR-03 protocol and fully centralized in the ALL-HR-11 protocol, using the EuroFlow approach [8]. Overall, complete remission (CR) was achieved in 58/62 cases (94%), 80% and 60% of whom showed MRD levels after induction ≤0.1% and ≤0.01%, respectively; 13/58 (22%) of patients were transplanted in first CR (Supplemental Table S4).

DNA from adult T-ALL bone marrow (BM) samples was obtained from the ALL research group collection (registered number: 2014999E000809), the Spanish National DNA Bank Carlos III (PT13/0001/0037 and PT13/0010/0067), La Fe Bio-bank (PT13/0010/0026) and collections from several PETHEMA hospitals. Samples were obtained in accordance with the principles of the Declaration of Helsinki and the Spanish legislation for protection of personal data and research on human samples, after patients provided their written informed consent. The study was approved by the Institutional Review Board of the Hospital Germans Trias i Pujol (Badalona, Spain).

Genomic qPCR assays

qPCR assays were performed in a Light-Cycler®480 Real-time PCR system with the SYBR Green Master Mix chemistry (Roche Diagnostics, Mannheim, Germany), using primer sequences for the *CDKN2A/ARF* (exon 3), *CDKN2B* (exon 1) genes. Most alterations reported so far in cancer patients that involve the *CDKN2A* gene are accompanied by a parallel loss of

function of the *ARF* gene, due to the unusual structure of this gene locus. This prompted us to use primers that anneal on exon 3 of the *CDKN2A* gene which is also shared by the *ARF* gene. Reference genes (59KB and L1PA), and PCR conditions reported elsewhere [9-10]. In case of trisomy 8, the L1PA together with the L1 (Terribas *et al.* manuscript in preparation) primers were used. Each reaction included 8 ng of DNA template, 5 µl 2Xmaster mix, 1 µmol/L of each primer in a total volume of 10 µl. qPCR was performed in triplicate for each primer set and sample. Each set of PCR assays included both a negative control without template and a calibrator sample with a diploid (2N) DNA content. For qPCR data analysis, a previously published method [9] based on qBase relative quantification, was used. Relative copy number (RCN) values were adjusted to the amount of normal residual DNA content (2N), as follows: DNA with no copies (0N) for the CD*KN2B/ARF/CDKN2A* locus (sample containing 100% blasts) was mixed with different amounts of normal DNA (2N) to obtain serial dilutions containing 80%, 60%, 40% and 20% of blasts DNA, and assessed by qPCR. A similar mixture with a pure heterozygous (1N) sample, was also made and analyzed in parallel. In both cases, the RCN values obtained displayed a linear correlation with the percentage of blasts (Supplemental Figure S3; A-B). Then, the RCN value corresponding to the percentage of contaminant cells was subtracted from the RCN value obtained for each individual sample, in case of contamination. To establish which linear regression curve should be applied per case, a cut-off value was defined to distinguish mono-allelic from bi-allelic deletions, using the mean RCN value of selected (pure) blast cell samples (Supplemental Table S5; A-B).

SNP-array studies.

CNA were screened by CytoScan high-density arrays (Affymetrix, Santa Clara, CA) according to the manufacturer's instructions (average probe spacing of 700bp in exonic regions). CNA were detected and analyzed with the Chromosome Analysis Suite (ChAS 3.1) software provided by the manufacturer. CNA identified by <8 probes and <1Kb were not considered. Constitutional CN polymorphisms were excluded by filtering the data using the Affymetrix data on healthy controls DNA from 2,700 anonymous individuals across the globe), and the Database of Genomic Variants (http://dgv.tcag.ca/dgv/app/home).

Interphase fluorescence *in situ* hybridization (FISH) studies

Interphase FISH analyses were performed on Carnoy fixed single cell suspensions made at the participating centers. FISH was carried out with the LSI CDKN2A/CEP9 probe (Abbott, Santa Clara, CA) and a home-made bacterial artificial chromosome (BAC) probe (CHORI collection, RP11-467K20 and RP11-625H1), according to the manufacturer's instructions. Per sample, ≥100 interphase nuclei were scored and a cut-off identified with the locus-specific probe of >5%, was used to define deletions in the *CDKN2A/ARF/CDKN2B* gene locus.

Statistical analyses

Quantitative variables were expressed as median (range), while frequencies were used for qualitative variables. For comparisons among groups (for categorical variables), the Chi-square or Fisher exact tests were used. OS was determined from the time of diagnosis to death or the last follow-up visit and OS curves were plotted by the Kaplan-Meier method; the log-rank test was used to evaluate the statistical significance of differences between OS curves. Multivariate analysis for OS was performed by the Cox proportional hazards regression model. All statistical analyses were carried out with the SPSS (Statistical Package for Social Sciences) package v24.0 (IBM, Armonk, NY) and the R 3.3.2 software (https://www.ibm.com/es-es/marketplace/spss-statistics). For all statistical analyses; the level of significance was set at P<.05.

Online Supplemental Table legends

Online Supplemental Table S1. Frequency and type of *CDKN2A/ARF/CDKN2B* gene deletions as detected by qPCR in adult T-ALL (n=64).

Online Supplemental Table S2. Comparison between the CNA status of the *CDKN2A/ARF* and *CDKN2B* genes in adult T-ALL as assessed by qPCR, SNP-array and iFISH techniques.

Online Supplemental Table S3. Adult T-ALL: prognostic factors for overall survival.

Online Supplemental Table S4. Adult T-ALL patient characteristics at diagnosis and follow-up.

Online Supplemental Table S5. (A) RCN values obtained for the *CDKN2A/ARF* and *CDKN2B* genes in selected samples with a 100% blast cell content. (B). Most robust cut-off values to distinguish between normal, heterozygous and homozygous genotypes. The mean and standard deviation (SD) of the values obtained in panel A are indicated for each genotype.

Online Supplemental Figure legends

Online Supplemental Figure S1. Prognostic impact of the *CDKN2B* gene CNA status on overall survival of adult T-ALL patients (n=62). In panel A all *CDKN2B* gene deletions were analyzed together, while in panel B bi-allelic and mono-allelic *CDKN2B* gene deletions were separately considered.

Online Supplemental Figure S2. Flowchart summarizing the HR-20011 PETHEMA treatment protocol, including the time points at which MRD assessment was performed (highlighted in red).

Online Supplemental Figure S3. Calibration curves used to calculate RCN values according to the different percentage contamination of the sample by normal (i.e. non-blastic) cells. In panel A, a pure (100% blasts) homozygous sample was mixed with different amounts of normal (2N) DNA, as shown on the x-axis. RCN values are shown on the y-axis. In panel B a pure (100%) heterozygous sample was mixed with different amounts of normal (2N) DNA, as shown on the x-axis; RCN values are depicted on the y-axis.

Table S1

	Bi-allelic deletion	Mono-allelic deletion	No-allelic deletion	Bi+mono deletion
CDKN2A/ARF	26/64 (41%)	6/64 (9%)	32/64 (50%)	32/64 (50%)
CDKN2B	22/64 (34%)	8/64 (12,5%)	34/64 (53%)	30/64 (47%)
CDKN2A/ARF/CDKN2B	20/64 (31%)	2/64 (3%)	29/64 (45%)	22/64 (34%)

13/64 cases (20%) presented distinct CNA status for the *CDKN2A/ARF* and *CDKN2B* genes.

Table S2

Sample ID	% Blasts	CNA status (qPCR)		CNA status (SPN array)		iFISH results
		CDKN2A/ARF	*CDKN2B*	*CDKN2A/ARF*	*CDKN2B*	*CDKN2A/ARF/CDKN2B*
023T/D	100	1	0	1	0	NE
028T/D	100	0	0	0	0	bi-allelic deletion
AAA1	99	0	0	0	0	bi-allelic deletion
AAA5	100	2	2	2	2	NE
AA17	96	0	1	0	1	NE
AA21	84	0	2	0	2	NE
AA33	43	0	0	0	0	NE
AA36	60	1	0	1	0	NE
AA39	98	0	0	0	0	bi-allelic deletion
AA45	98	0	1	0	1	NE
AA54	100	0	0	0	0	NE
AA78	95	0	0	0	0	bi-allelic deletion **
AA81	90	0	0	0	0	NE
AAA7	95	0	0	0	0	NE
027T7D	100	2	2	2	2	NE
AA19	70	2	2	2	2	no deletion
AA24	70	0	0	NE	NE	bi-allelic deletion
021T/D	100	2	2	2	2	NE
AA42	87	2	2	2	2	NE
AA44	44	2	2	2	2	NE
AA47	92	2	2	2	2	NE
036T/D	100	2	2	2	2	NE
AA85	70	2	2	2	2	NE
AA58	29	2	2	2	2	NE
AA60	72	0	2	0	2	NE
AA82	99	2	2	2	2	NE
AA86	79	2	2	2	2	NE
AA88	90	0	2	0	2	NE
AA90	74	0	0	0	0	NE
054T/D	94	2	2	2	2	no deletion
055T/D	72	0	0	0	0	bi-allelic deletion
AA94	100	2	2	2	2	no deletion
AA97	100	2	2	2	2	no deletion
A100	100	2	2	2	2	NE

0 corresponds to bi-allelic gene deletions; 1 to mono-allelic gene deletions and 2 to no gene deletion (normal genotype)
NE= not evaluated
** home-made probe (CHORI BAC collection)

Figure S1

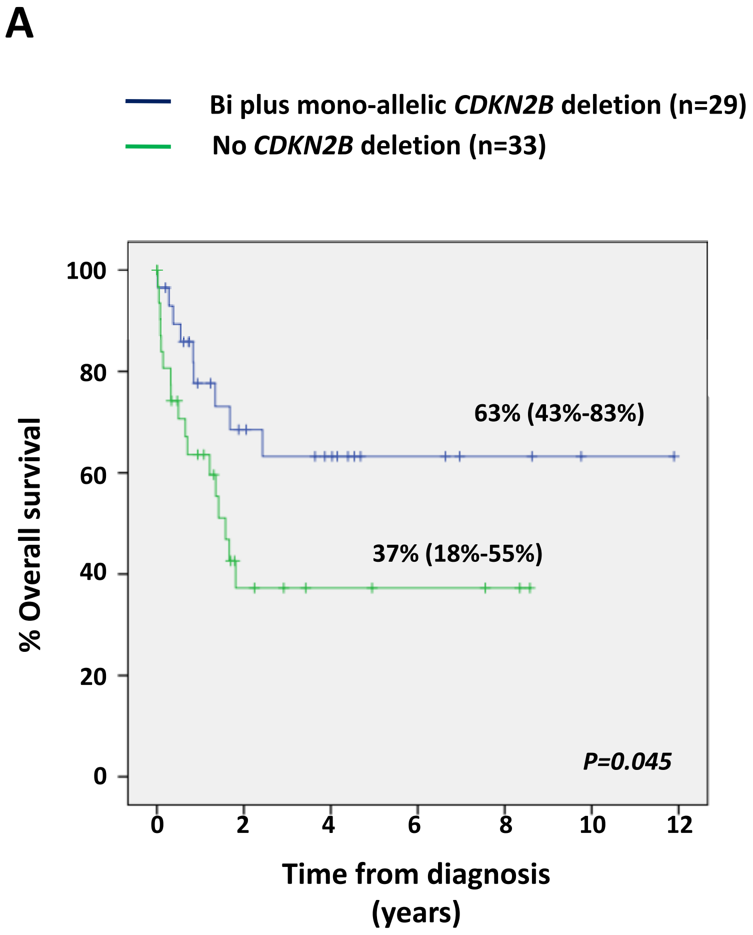
A
Bi plus mono-allelic CDKN2B deletion (n=29)
No CDKN2B deletion (n=33)
100
80
60
40
20
0
% Overall survival
63% (43%-83%)
37% (18%-55%)
P=0.045
0
2
4
6
8
10
12
Time from diagnosis
(years)

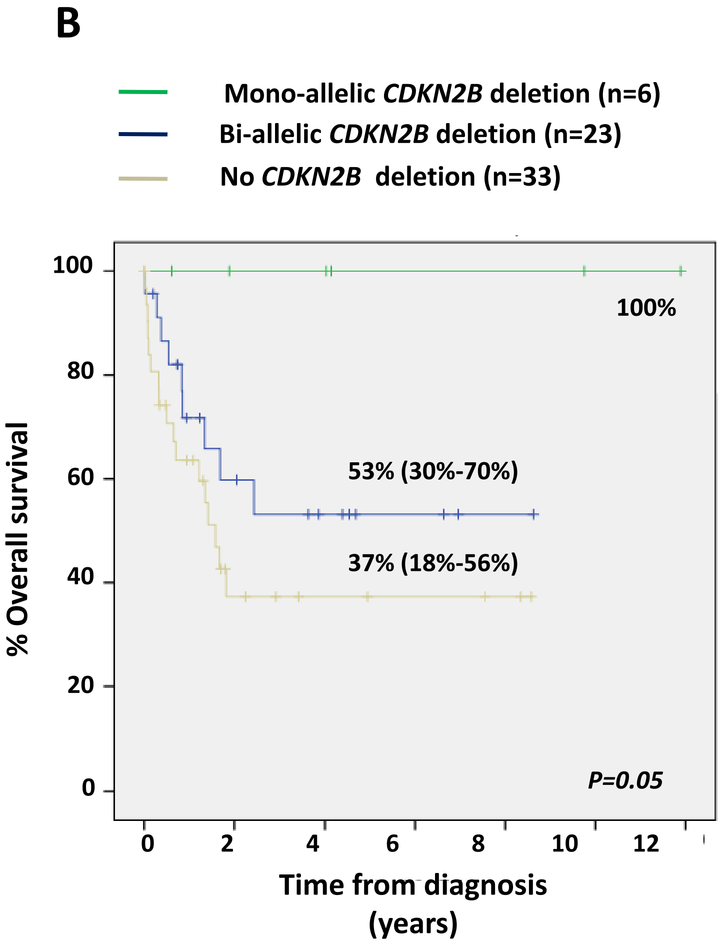
B
Mono-allelic CDKN2B deletion (n=6)
Bi-allelic CDKN2B deletion (n=23)
No CDKN2B deletion (n=33)
100
80
60
40
20
0
% Overall survival
100%
53% (30%-70%)
37% (18%-56%)
P=0.05
0
2
4
6
8
10
12
Time from diagnosis
(years)

Table S3

	N	Univariate analysis		Multivariate analysis	
		**HR (95% CI)**	**P-value**	**HR (95% CI)**	**P-value**
**Non deleted CDKN2A/ARF**	30/62		NS		
**Non deleted CDKN2B**	33/62	2.25 (0.99 ; 5.06)	0.05		NS
**Non deleted CDKN2A/ARF/CDKN2B**	28/62		NS		
**Immunophenotype:**			0.04		NS
**ETP-ALL vs other immunophenotype**	12/59	3.74 (1.32 ; 10.59)	0.01		
**MRD ≥0.1% after first induction**	11/55	3.04 (1.20 ; 7.73)	0.02	3.08 (1.19 ; 7.94)	0.02

HR: hazard ratio; N: number of cases; NS: statistically not significant

Figure S2

PETHEMA HR2011-FLOW CHART

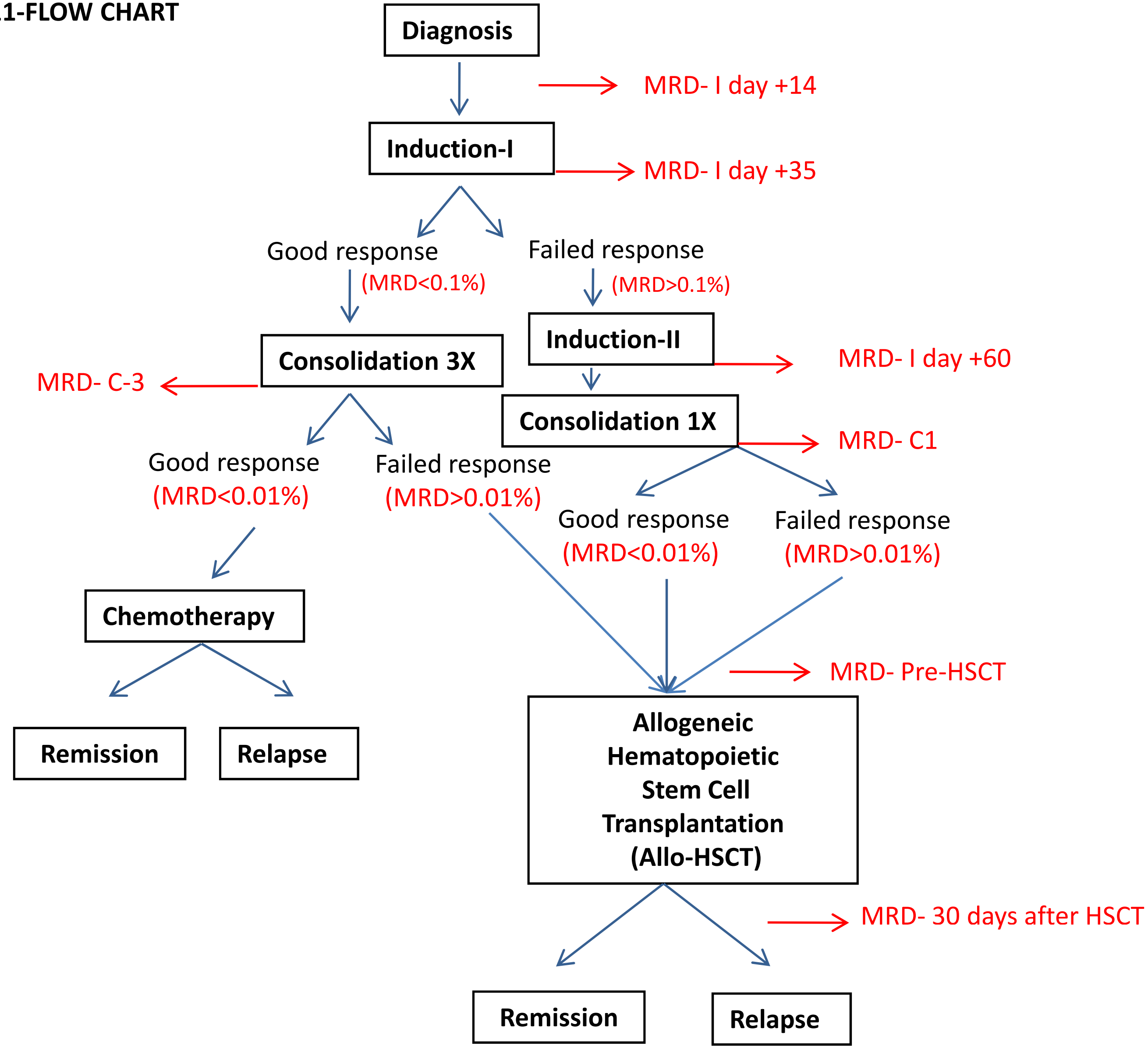

Table S4

**Patient characteristics at diagnosis and follow-up**	
Age (years)[range]	36 [16- 72]
Gender, F/M (%)	19 (31%)/ 43 (69%)
**Disease related features**	
WBC count (x 10^9/L)	26.5 (0.6-431)
CNS involvement (Y/N/unknown)	6/ 55/ 1 (10%)
Overall cytogenetics:	
Complex karyotypes*	3 (5%)
No complex	49 (79%)
Unknown	10 (16%)
Cytogenetic Subsets:	
del(6q)	5 (9%)
TLX3 t(5;7)(q35;V)	2 (4%)
TLX1 t(10;14)(q24;q11)	1 (2%)
TAL1 del(1)(p22p32)	1 (2%)
LMO1 t(11;14)(p15;q11)	1 (2%)
KMT2A-ENL t(11;19)(q23;p13)	1 (2%)
CCND2 t(12;14)(p13.3;q11.2)	1 (2%)
Immunophenotypic subtype:	
ETP-ALL	12 (20%)
Pre-T/T II	16 (26%)
Cortical/T III	19 (31%)
Mature/T IV	12 (20%)
Unknown	3 (5%)
**Treatment-related disease feaures**	
CR (Y/N)	58/ 4 (94%)
MRD <0.1% at CR (Y/N/unknown)	44/ 11/ 7 (80%)
MRD <0.01% at CR (Y/N/unknown)	33/ 22/ 7 (60%)
Transplanted at first CR (Y/N)	13/ 45 (22%)

Results expressed as number of cases (percentage) or as median (range)
M: male; F: female; MRD: minimal residual diseases; Y: yes; N: no; CR: complete remission.
* ≥5 cytogenetic alterations

Figure S3

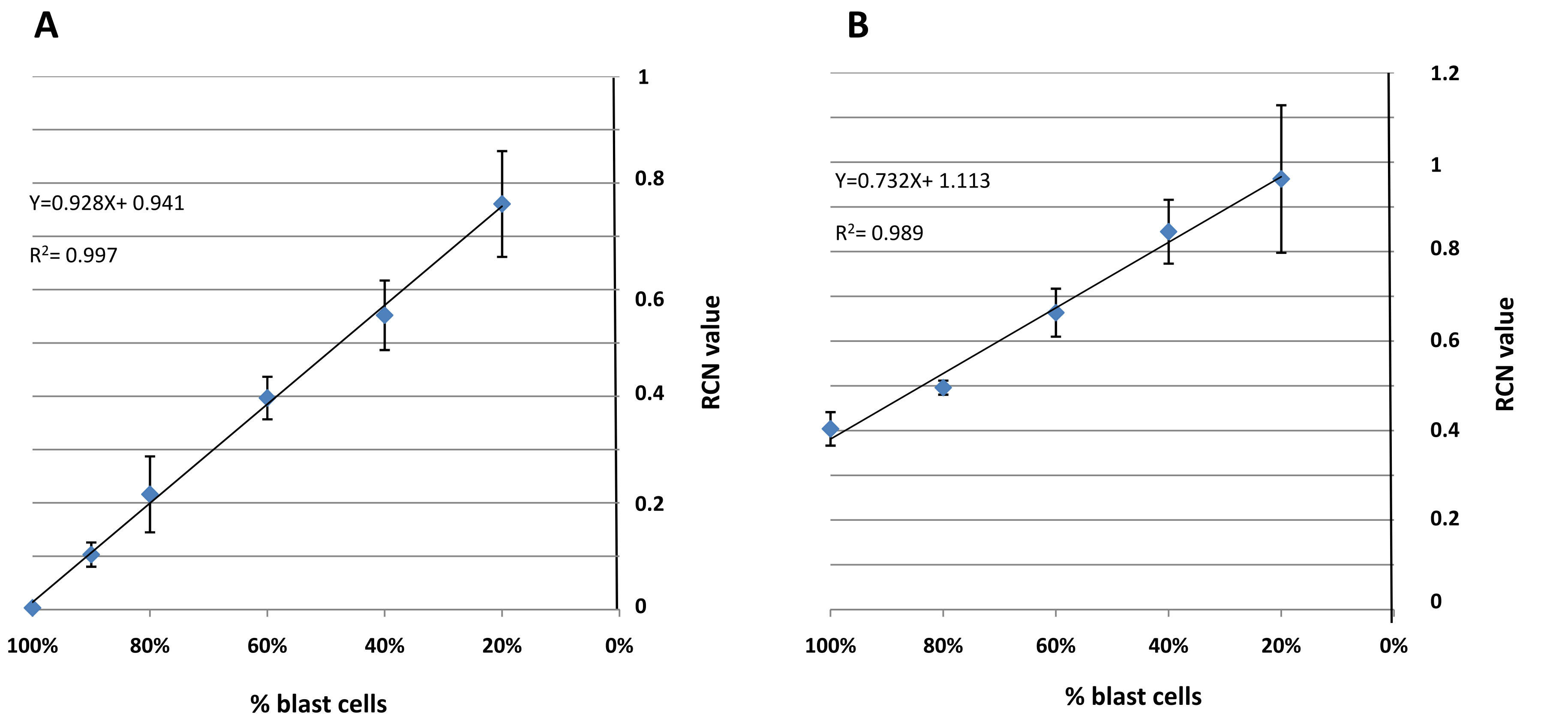
A
Y=0.928X+ 0.941
R2= 0.997
1
0.8
0.6
0.4
0.2
0
RCN value
100%
80%
60%
40%
20%
0%
% blast cells
B
Y=0.732X+ 1.113
R2= 0.989
1.2
1
0.8
0.6
0.4
0.2
0
RCN value
100%
80%
60%
40%
20%
0%
% blast cells

Table S5

A

Sample ID	*CDKN2A/ARF RCN*	*CDKN2B RCN*
023T/D	0.5	0.0
028T/D	0.0	0.0
AA54	0.1	0.1
009T/D	1.5	1.4
027T7D	1.6	1.7
021T/D	1.2	1.5
036T/D	1.4	1.2
AA18	0.5	1.5
AA51	0.0	0.0
AA70	1.3	1.4
A100	0.9	0.6
A104	1.3	1.1
A101	0.2	0.1
A103	1.4	1.1
A111	1.1	1.3
AA76	0.0	0.0
AA94	0.8	0.9
A118	0.2	0.1
A102	1.0	0.9

B

	CDKN2A/ARF/CDKN2B locus RCN (mean ±SD)
**No-allelic deletions (n=22)**	
Mean ±SD	1.25 ±0.23
Mean-2 SD	**0.8**
**Mono-allelic deletions (n=3)**	
Mean ±SD	0.53 ±0.08
Mean-2 SD	**0.4**
**Bi-allelic deletions (n=13)**	
Mean ±SD	0.06 ±0.06
Mean-2 SD	-0.1
